# Methylglyoxal Acts as a Tumor-Promoting Factor in Anaplastic Thyroid Cancer

**DOI:** 10.3390/cells8060547

**Published:** 2019-06-06

**Authors:** Cinzia Antognelli, Sonia Moretti, Roberta Frosini, Efisio Puxeddu, Angelo Sidoni, Vincenzo N. Talesa

**Affiliations:** 1 Department of Experimental Medicine, University of Perugia, Piazza Lucio Severi 1, 06132 Perugia, Italy; roberta.frosini@unipg.it (R.F.); angelo.sidoni@unipg.it (A.S.); vincenzo.talesa@unipg.it (V.N.T.); 2 Department of Medicine, University of Perugia, Piazza Lucio Severi 1, 06132 Perugia, Italy; sonia.moretti@unipg.it (S.M.); efisio.puxeddu@unipg.it (E.P.)

**Keywords:** methylglyoxal, anaplastic thyroid cancer, glyoxalase 1, EMT, AGEs, resveratrol, aminoguanidine

## Abstract

Methylglyoxal (MG) is a potent inducer of advanced glycation end products (AGEs). MG, long considered a highly cytotoxic molecule with potential anticancer value, is now being re-evaluated to a protumorigenic agent in some malignancies. Anaplastic thyroid cancer (ATC) is an extremely aggressive and highly lethal cancer for which conventional therapies have proved ineffective. Successful therapeutic intervention in ATC is undermined by our poor understanding of its molecular etiology. In the attempt to understand the role of MG in ATC aggressiveness, we used immunohistochemistry to examine the level of MG protein adducts in ATC and slow-growing papillary thyroid cancer (PTC). We detected a high level of MG adducts in ATC compared to PTC ones, suggesting a protumor role for MG-mediated dicarbonyl stress in ATC. Accordingly, MG adduct accumulation in ATC cells in vitro was associated with a marked mesenchymal phenotype and increased migration/invasion, which were both reversed by aminoguanidine (AG)—a scavenger of MG—and resveratrol—an activator of Glyoxalase 1 (Glo1), the key metabolizing enzyme of MG. Our study represents the first demonstration that MG, via AGEs, acts as a tumor-promoting factor in ATC and suggests that MG scavengers and/or Glo1 activators merit investigations as potential therapeutic strategies for this malignancy.

## 1. Introduction

Methylglyoxal (MG) is a dicarbonyl compound with a low molecular mass mainly generated in cells through the spontaneous degradation of triose phosphate intermediates of glycolysis [1]. MG is a powerful glycating agent and is much more reactive than parent glucose in glycation processes. MG glycation reactions result in the production of advanced glycation end products (AGEs), inducing dicarbonyl stress and cellular damage [2]. MG-derived dicarbonyl adducts exert complex pleiotropic effects on normal and pathologic processes in cells, including modulation of protein biological activity [3] and stability [4], and generation of reactive oxygen species (ROS) and oxidative stress [5], which may culminate in distinct biological outcomes [6,7,8,9,10,11]. In particular, supra-physiological accumulation of hydroimidazolone (MG-H1) and argpyrimidine (AP), AGEs originating from MG-mediated post-translational modification of proteins at arginine residues, has been shown to induce oxidative DNA damage and apoptosis [6,9,10,11]. However, post-translational modifications by MG-derived AP can also enhance the functionality of fundamental stress-inducible proteins implicated in cellular recovery after exposure to damaging stimuli and protection against apoptosis, including heat shock protein 27 (Hsp27), thus playing an important role in cell survival [12,13]. Furthermore, there is also evidence that some MG-derived AGEs, including AP, are endowed with antioxidant properties [14]. These apparently divergent functions imply that MG, like other reactive species, may exert different or even opposite biological effects, depending on its levels [15] and the cellular context. Intracellular accumulation of MG is limited by the ubiquitous glutathione-dependent enzyme, Glyoxalase 1 (Glo1) [1]. In the oncologic ambit, MG has long and consistently been considered a potential anticancer agent [1], exerting a highly cytotoxic effect. Indeed, Glo1 is frequently overexpressed in many human malignancies as a survival defense strategy against MG cytotoxicity [1,16,17,18,19,20]. The overexpression of Glo1 in tumor cells makes this enzyme a pivotal target for anticancer drug development, and, in fact, Glo1 inhibitors, alone or in combination with other drugs, have offered potential benefit in the management of some human malignancies [21,22,23]. However, recent evidence suggests that MG and MG-induced carbonyl stress display cancer-promoting properties, with a major role in colorectal cancer progression [24,25,26]. Hence, the ambivalent role of MG, and consequently, of its major scavenging enzyme Glo1, seem to turn out more and more cancer type-dependent and it is expectable that cancer tissues and cell lines with different molecular backgrounds and MG detoxification rates will respond differently to MG stress [24]. Anaplastic thyroid carcinoma (ATC) is an extremely aggressive malignancy with undifferentiated features, for which conventional treatments are usually not effective [27]. Early tumor dissemination results in 20–50% of patients having distant metastases and 90% having adjacent tissue invasion on presentation [28]. Therefore, although ATC represents less than 2% of all thyroid cancers (TCs), it accounts for around 50% of TC mortality [29]. Successful therapeutic intervention in ATC is undermined by our poor understanding of its molecular etiology. The present study aimed to investigate the importance of MG-induced dicarbonyl stress and of Glo1 in the context of ATC. Specifically, we showed that high tumor MG adduct accumulation was associated with low Glo1 activity levels and aggressiveness of ATC patients. In accordance with these observations, in vitro experiments using appropriate ATC cell models showed increased invasion/migration properties associated with MG adduct accumulation that could be reverted by aminoguanidine (AG), a scavenger of MG, and resveratrol, an activator of Glo1. To our knowledge, our study represents the first demonstration of a correlation between dicarbonyl stress and ATC aggressiveness and suggests that blockade of dicarbonyl stress by MG scavengers and/or Glo1 activators merit investigations as a potential new therapeutic strategy for the treatment of this lethal disease.

## 2. Materials and Methods

### 2.1. Reagents

S-p-Bromobenzylglutathione cyclopentyl diester (BBGC) (cod. SML1306), resveratrol (cod. R5010), aminoguanidine bicarbonate (AG) (cat. 396494), IL1 receptor antagonist (cat. SRP3327), and methylglyoxal (MG) (cat. M0252) were purchased from Merck Spa (Milan, Italy). We excluded the presence of significant formaldehyde contamination (<3%) in the lot of MG by nuclear magnetic resonance (NMR) analysis. Recombinant human TGF-β1 (cat. 240-B-002) was from R&D Systems, (Bio-techne S.r.l., Milan, Italy). Recombinant human IL-1β (cat. 200-01B) was from DBA Italia S.r.l. (Milan, Italy). BBGC and resveratrol were dissolved in dimethyl sulfoxide (DMSO, Merck Spa, Milan, Italy) (final DMSO concentration in incubations = 0.01%).

### 2.2. Patient Samples and Cell Lines

Tissue samples for immunohistochemical analysis were obtained from the archive of the Pathology Division of the University of Perugia. Tissue extracts and cDNA from tissue samples were previously obtained [30]. Genetic analysis was performed as previously described [30]. The study was designed and performed according to the Declaration of Helsinki and the protocol was approved by the local medical Ethics Committee. Each study participant provided written informed consent. Characteristics of patients are summarized in Table 1.

The B-CPAP cell line (derived from a papillary TC, PTC) was acquired from Leibniz Institute DSMZ (Braunschweig, Germany) and grown in RPMI 1640 supplemented with 10% fetal bovine serum (FBS, ThermoFisher Scientific, Monza, Italy) [30]. The TPC1 cell line (derived from a PTC) was provided by Professor Alfredo Fusco (University of Naples, Naples, Italy) and was grown in DMEM supplemented with 10% FBS; authentication included detection of ret/PTC1 [30]. The 8505C cell line (derived from an ATC) was provided by Dr. Carmelo Nucera (Harvard Medical School, Boston, MA, USA) and was grown in RPMI 1640 supplemented with 10% FBS; authentication was conducted by DNA profiling at the University of Colorado Cancer Center DNA Sequencing and Analysis Core (Aurora, CO, USA) [30]. The CAL62 cell line (derived from an ATC) was acquired from Leibniz Institute DSMZ and grown in DMEM supplemented with 10% FBS [30].

### 2.3. Immunohistochemistry (IHC) and Evaluation of Immunohistochemical Staining

Immunohistochemistry (IHC) was performed as previously described [31,32]. Briefly, formalin-fixed and paraffin-embedded sections that were four micrometers thick were deparaffinized in xylene and rehydrated. Immunohistochemical staining of the sections was performed with the Bond III Leica automated immunostainer (Leica Biosystems, Newcastle Ltd., Newcastle upon Tyne, UK) using the kit Bond™ Polymer Refine Detection (Leica Biosystems, Newcastle Ltd.). Sections were incubated with anti-MG-H1 (cat. STA-011, diluted 1:50, 3D11 clone, DBA Italia S.r.l.) or anti-Glo1 (cat. sc-133144, diluted 1:4500, DBA Italia S.r.l.) antibodies for 15 min with a retrieval of 20 min at pH = 6. Slides were counterstained with hematoxylin. The immunohistochemically stained sections were evaluated by an experienced anatomopathologist (AS). Scoring of the staining was done according to the intensity of the staining (0, 1+, 2+, and 3+) and the percentage of positive cells (0–25%, 25–50%, 50–75%, and 75–100%). The results obtained with the two scales were multiplied together, as described in [33], yielding a single scale with steps of 0, 1+, 2+, 3+, 4+, 6+, and 9+; where 0, 1+, and 2+ were considered to be negative or weak staining; while 3+, 4+, 6+, and 9+ were considered to be medium (moderate) or strong (intense) staining.

### 2.4. Detection of Methylglyoxal (MG)-H1 Protein Adducts

MG-H1 protein adducts were measured by using a competitive enzyme-linked immunosorbent assay (ELISA) kit (cat. STA-811, DBA Italia S.r.l.). Briefly, MG-conjugate was coated on the ELISA plate as specified by the manufacturer. Samples or MG–BSA standards were added in triplicate to the preadsorbed plate. An anti-MG-specific monoclonal antibody was incubated for 1 h at room temperature, followed by washes and an incubation with horseradish peroxidase (HRP)-conjugated secondary antibody, as recommended by the kit’s manufacturer. The contents of MG-H1 adducts in the protein samples were determined through a 4P-logistic regression equation by comparing the absorbance at 450 nm with that of the MG–BSA standard curve. A Mindray MR-96A Microplate Reader (Mindray Medical Italy S.r.l., Milan, Italy) was used for readings.

### 2.5. RNA Isolation, Reverse Transcription, and Real-Time Reverse Transcriptase-Polymerase Chain Reaction (RT-PCR) Analyses

Total cellular RNA was isolated using TRIzol Reagent (cat. 15596026, ThermoFisher Scientific). cDNA was then synthesized from 1 μg of RNA with the RevertAid™ H Minus First Strand cDNA Synthesis Kit (cat. K1632, ThermoFisher Scientific). Gene expression versus β-actin was evaluated by RT-PCR on a MX3000P Real-Time PCR System (Agilent Technology, Milan, Italy). The sequences of the oligonucleotide primers are reported in Table 2. PCR primers were designed using Beacon Designer 4 software (version 4.0, Agilent Technology) from published sequence data stored in the NCBI database. PCR reactions were performed in a total volume of 20 μL, which contained 25 ng of cDNA, 1X Brilliant II SYBR^®^ Green QPCR Master Mix (cat. 600828, Agilent Technology), ROX Reference Dye (cat. 600804, Agilent Technology), and 600 nM of specific primers. The thermal cycling conditions were 1 cycle at 95 °C for 5 min followed by 45 cycles at 95 °C for 20 s and 60 °C for 30 s. In order to verify the possible coamplification of unspecific targets, melting curves were performed for all of the primer pairs in standard conditions. The data required for carrying out a comparative analysis of gene expression were obtained by means of the 2−(ΔΔCT) method [34].

### 2.6. Cell Lysate Preparation for SDS-PAGE

Total protein extraction was performed by lysing subconfluent cells with precooled radio-immunoprecipitation assay (RIPA) lysis buffer (cat. 89900, ThermoFisher Scientific) enriched with Halt Protease Inhibitor Cocktail (cat. 78430, ThermoFisher Scientific) and Halt Phosphatase Inhibitor Cocktail (cat. 78420, ThermoFisher Scientific) according to the manufacturer’s instructions. Protein concentration was determined with a bicinchoninic acid (BCA) kit (cat. 23225, ThermoFisher Scientific) with bovine serum albumin as a standard.

### 2.7. Nuclear Extract Preparation

For nuclear extracts, a FractionPREP Cell Fractionation kit (Biovision, Vinci-Biochem, Florence, Italy) was used.

### 2.8. SDS-PAGE and Western Blot Analyses

Sodium Dodecyl Sulphate-PolyAcrylamide Gel Electrophoresis (SDS-PAGE) and Western blot analyses were performed as previously described [10]. Briefly, samples of equal protein concentrations (20–40 µg) were treated with Laemmli buffer, boiled for 5 min, resolved on a 4–20% SDS-PAGE, and then blotted onto a nitrocellulose membrane by the iBlot Dry Blotting System (ThermoFisher Scientific). Nonspecific binding sites were blocked in Roti-Block (cat. A151.1, Prodotti Gianni S.r.l., Milan, Italy) for 1 h at room temperature and then incubated overnight at 4 °C with anti-Glo1 Ab (cat. sc-133144, diluted 1:1000, DBA Italia S.r.l.). After being washed with Tris-buffered saline/Tween, antigen–antibody complexes were detected by incubation of the membranes for 1 h at room temperature with the peroxidase-conjugated antimouse secondary antibody (cat. A9044; dil. 1:10,000, Merck Spa, Milan, Italy) and visualized by the ECL system (cat. WBKLS0500, Merck Spa, Milan, Italy). As internal loading controls, all membranes were subsequently stripped off the first Ab in a stripping buffer (100 mmol/L 2-mercaptoethanol, 2% SDS, and 62.5 mmol/L Tris-HCl; pH 6.8) and reprobed with the housekeeping Ab anti-β-actin (cat. sc-376421, diluted 1:000, DBA Italia S.r.l.).

### 2.9. Glo1 Enzyme Activity Assessment

Subconfluent cells were lysed in 100 mM KH_2_PO_4_, 1.5 mM dithiotreitol (DTT), and 1 mM ethylenediaminetetraacetic acid (EDTA) (pH 7) extraction buffer. Cell suspensions were homogenized and centrifuged at 16,000× *g* for 30 min at 4 °C. Protein extracts were used for enzyme activity measurement and for quantification of total protein concentration by using a BCA kit (cat. 23225, ThermoFisher Scientific) with bovine serum albumin as a standard. Glo1 enzyme activity was assayed by an established method [35]. Briefly, the assay solution contained 0.1 mol/L sodium phosphate buffer, pH 7.2, 2 mmol/L MG, and 1 mmol/L reduced glutathione (GSH). The reaction was monitored spectrophotometrically by following the increase in absorbance at 240 nm and 25 °C. One unit of activity was defined as 1 µmol of S-D lactoylglutathione produced per minute.

### 2.10. Transwell Migration and Invasion Assays

Transwell migration and invasion assays were carried out by using the commercially available CytoSelect™ 24-Well Cell Migration Assay (cat. CBA-100 DBA Italia S.r.l.) and CytoSelect™ 24-Well Cell Invasion Assay kits (cat. CBA-110, DBA Italia S.r.l.), respectively, according to the manufacturer’s instructions.

### 2.11. IL-1β, IL1R1, Phospho-IRAK-1, Phospho-TAK1, Phospho-IKK, p65 NF-kB, TGF-β1, and p-FAK Detection

IL-1β (cod. BMS224-2), TGF-β1 (cod. BMS249-4), and p-FAK (cod. KHO0441) were measured by using specific, commercially available ELISA kits all from ThermoFisher Scientific. IL1R1 (cod. KA2210) and p65 NF-kB (cod. ABIN965407) were measured by using specific, commercially available ELISA kits from DBA Italia S.r.l. Phospho-IRAK-1 (cod. OKAG01835) and phospho-IKK (cod. OKAG01971) were measured by using specific, commercially available ELISA kits from Aurogene (Rome, Italy). Phospho-TAK1 (cod. PEL-TAK1-S412-1) was measured by using the specific, commercially available ELISA kit Prodotti Gianni S.r.l. (Milan, Italy). All kits were used as per the manufacturer’s instructions.

### 2.12. Statistical Analysis

All data were generated from three independent experiments and expressed as means ± standard deviation (SD). One-way analysis of variance with Dunnett′s correction was used to assess differences among groups. Results from the immunohistochemical analysis were analyzed using Fisher’s exact test. Correlation analyses were carried out with the Spearman’s correlation test. Statistical significance was set at *p* ≤ 0.05.

## 3. Results

### 3.1. MG-H1 Adducts Accumulated in Anaplastic Thyroid Cancer (ATC) Tissues When Compared with Papillary Thyroid Cancer (PTC) Ones

**Figure 1:**
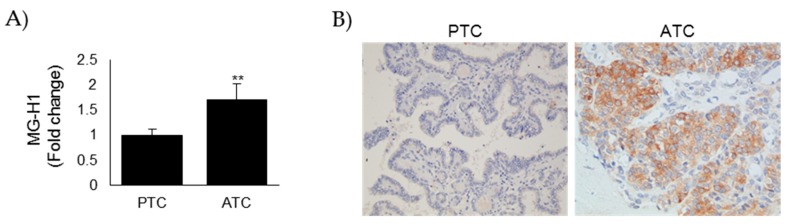
MG-derived hydroimidazolone (MG-H1) adducts are accumulated in anaplastic thyroid cancer (ATC) tissues when compared with papillary thyroid cancer (PTC) ones. (**A**) Intracellular levels of MG-H1, measured by a specific ELISA kit, in protein extracts from PTC (*n* = 5, #6–10) and ATC (*n* = 5, #1–5) tissues. Results are expressed as mean ± SD. ** *p* < 0.01; (**B**) Representative immunohistochemical staining of MG-H1 on PTC (*n* = 5, #16–20) and ATC (*n* = 5, #11–15) tissues (200× magnification).

MG is a potent protein glycating agent. Glycation is directed to guanidino groups of arginine residues forming mainly hydroimidazolone N (δ)-(5-hydro-5-methyl-4-imidazolon-2-yl)-ornithine (MG-H1) residues [36]. MG-H1 formation is damaging to the proteome as modification is often directed to functionally important arginine residues [36]. With an ELISA kit specific to MG-H1, we evaluated the intracellular levels of this adduct on protein extracts from 5 ATC (#1–5) and 5 PTC (#6–10) tissues. As shown in Figure 1A, MG modified proteins accumulated more in ATC protein extracts than in PTC ones, indicating that MG-mediated dicarbonyl stress was more elevated in aggressive thyroid cancer. A comparable result was obtained when we next examined the accumulation of MG-H1 on 5 ATC (#11–15) and 5 PTC (#16–20) tissues using IHC (Figure 1B). In fact, we found that MG-H1 staining was moderate/strong in ATC tumors, while it was negative/weak in PTC ones (Figure 1B) (importantly, all PTC exhibited a negative to weak level of MG-H1, while all ATC exhibited a moderate to strong staining), thus reinforcing the signature of MG-mediated dicarbonyl stress in ATC tumors and suggesting a protumor role of MG-H1 in this aggressive malignancy.

### 3.2. Glo1 Expression in ATC and PTC Samples

**Figure 2:**
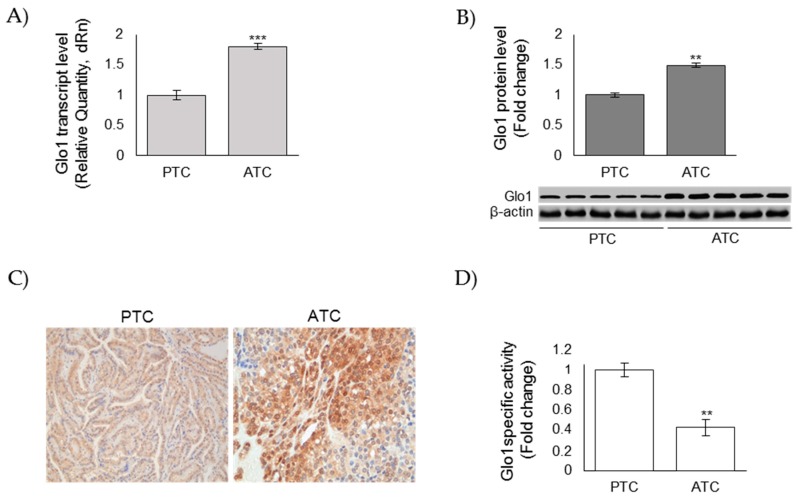
Glyoxalase 1 (Glo1) expression in anaplastic (ATC) and papillary (PTC) thyroid cancer samples. (**A**) Glo1 mRNA expression, evaluated by real-time PCR, after total RNA extraction from five ATC (#1–5) and five PTC samples (#6–10) and cDNA synthesis; (**B**) representative Western blot of Glo1 protein expression measured on lysates from five PTC (#6–10) and five ATC (#1–5) tissues. β-actin was used as loading control. The histogram, representing the densitometric analysis of the blots, indicates mean ± SD of all PTC (*n* = 5, #6–10) and ATC (*n* = 5, #1–5) samples analyzed; (**C**) representative immunohistochemical staining of Glo1 on PTC (*n* = 5, #16–20) and ATC (*n* = 5, #11–15) tissues (200× magnification); (**D**) Glo1 enzyme activity was measured in total protein extracts according to a spectrophotometric method, and increases in absorbance resulting from the formation of S-d-lactoylglutathione were monitored at 240 nm (see Materials and Methods). ** *p* < 0.01, *** *p* < 0.01.

MG is primarily metabolized to innocuous products by Glo1 that, thereby, protects the proteome, providing an enzymatic defense against MG-mediated glycation [36]. In order to investigate a possible mechanism by which MG-derived adducts accumulated in aggressive ATC, we next evaluated Glo1 mRNA levels by real-time PCR in 5 ATC (#1–5) and 5 PTC (#6–10). We also evaluated Glo1 protein levels in 5 ATC (#1–5) and 5 PTC (#6–10) protein extracts or histological sections (#11–15 ATC and #16–20 PTC) by western blot (WB) or IHC, respectively. Unexpectedly, Glo1 mRNA expression levels resulted significantly higher in ATC than in PTC samples (Figure 2A). Similarly, Glo1 protein levels were markedly higher in ATC than in PTC, both when detected by WB on protein extracts (Figure 2B) or by IHC on tissues (Figure 2C) (importantly, all PTC exhibited a negative to weak level of Glo1 expression, while all ATC exhibited a moderate to strong staining). Thus, we next evaluated protein extracts from ATC and PTC by spectrophotometric methods, and we found that Glo1-specific activity was significantly lower in ATC tumors than in PTC ones (Figure 2D). These results suggested that the accumulation of MG-mediated dicarbonyl stress observed in ATC tumors was consequent to a decreased functionality of Glo1.

### 3.3. Evaluation of MG-H1 and Glo1 Expression in Human Thyroid Cancer (TC) Cell Lines

**Figure 3:**
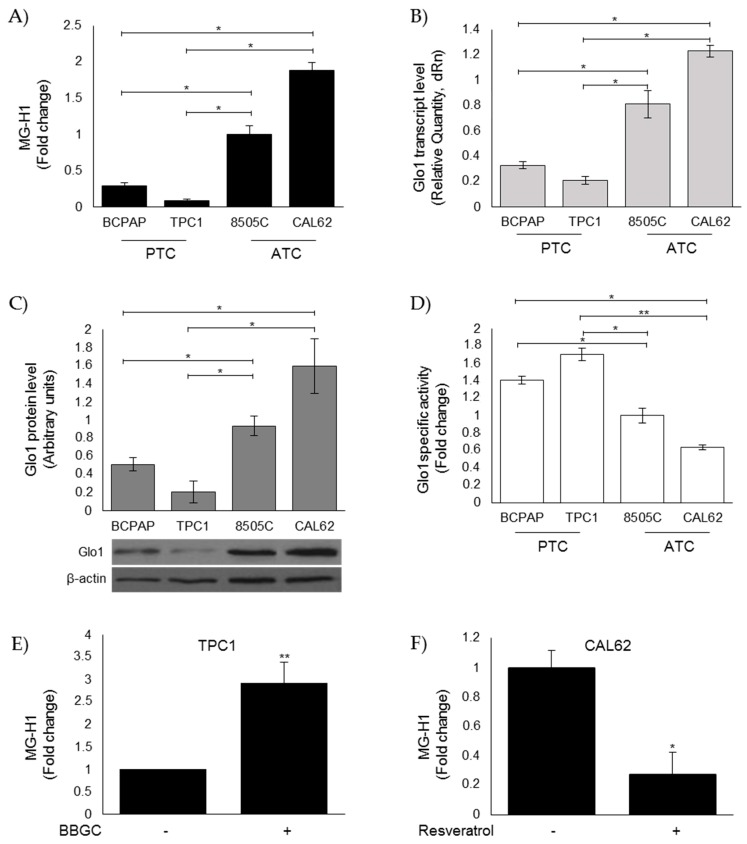
Evaluation of MG-H1 and Glo1 expression in human thyroid cancer (TC) cell lines. PTC (BCPAP and TPC1) and ATC (8505C and CAL62) cell models grown to confluence under standard conditions were lysed and analyzed by a specific ELISA kit (**A**), real-time PCR (**B**), Western blotting (**C**), and spectrophotometric enzymatic (**D**) assays, as described in the Materials and Methods section. (**A**) Intracellular levels of MG-H1, analyzed by a specific ELISA kit. The histogram indicates mean ± SD of three different cultures, and each was tested in triplicate. (**B**) Glo1 mRNA expression levels, analyzed in triplicate by real-time PCR and normalized to the amount of an internal control transcript (β-actin). Results are expressed as relative mRNA level units and represent the mean ± SD of n ≥ 3 independent, real-time PCR experiments. (**C**) Representative Western blot (WB) and quantitative histogram of the relative Glo1 protein expression levels. β-actin was used as internal loading control for WB normalization. The WB bands of Glo1 were quantified by a densitometric analysis, and normalized optical density values were expressed as relative protein level arbitrary units. (**D**) Glo1 enzyme activity was measured in total protein extracts according to a spectrophotometric method, and increases in absorbance resulting from the formation of S-D-lactoylglutathione were monitored at 240 nm (see Materials and Methods). Results represent the mean (± SD) of n ≥ 3 independent experiments performed in triplicate. Intracellular levels of MG-H1 in the presence of (**E**) 1 µM Glo1 inhibitor bromobenzylglutathione cyclopentyl diester (BBGC) or (**F**) 50 µM Glo1 activator Resveratrol were analyzed for 48 h with a specific ELISA kit. The histogram indicates mean ± SD of three different cultures, and each was tested in triplicate. * *p* < 0.05; ** *p* < 0.01.

Next, we investigated MG-H1 intracellular levels and Glo1 expression in CAL62, 8505C, B-CPAP, and TPC1 human TC cell lines. In accordance with the in vivo results, we found significantly higher levels of MG-H1 in CAL62 and 8505C cells derived from ATCs [30] than in B-CPAP and TPC1 cells derived from PTCs [30] (Figure 3A). Glo1 expression significantly increased, at both transcript (Figure 3B) and protein (Figure 3C) levels, while expression decreased at the functional level (Figure 3D) in ATC cell models compared to the PTC ones. Interestingly, Glo1 enzyme activity in TC cells mirrored MG-mediated dicarbonyl stress. Specifically, the lowest Glo1 enzymatic activity was observed in CAL65 ATC cells (Figure 3D) where the highest MG-H1 intracellular accumulation was present (Figure 3A), and the highest Glo1 enzymatic activity was observed in TPC1 PTC cells (Figure 3D) where the lowest MG-H1 intracellular accumulation was present (Figure 3A), thus reinforcing the hypothesis of a mechanism by which MG-modified proteins accumulate in ATC as a consequence of Glo1 reduced functionality [6,31]. Indeed, Glo1 inhibition by BBGC [37] in TPC1 cells (Figure 3E) as well as Glo1 activation by resveratrol [38] in CAL62 cells (Figure 3F) in the PTC and ATC cell models, respectively, where the major changes were observed, confirmed our hypothesis. The biochemical evidence of BBGC and resveratrol effectiveness on Glo1 enzyme activity is shown in Figure S1. Altogether, these results suggested a role of the Glo1/MG-H1 axis in highly aggressive ATC cells.

### 3.4. MG Sustains the Aggressive Phenotype of ATC CAL62 Cells

**Figure 4:**
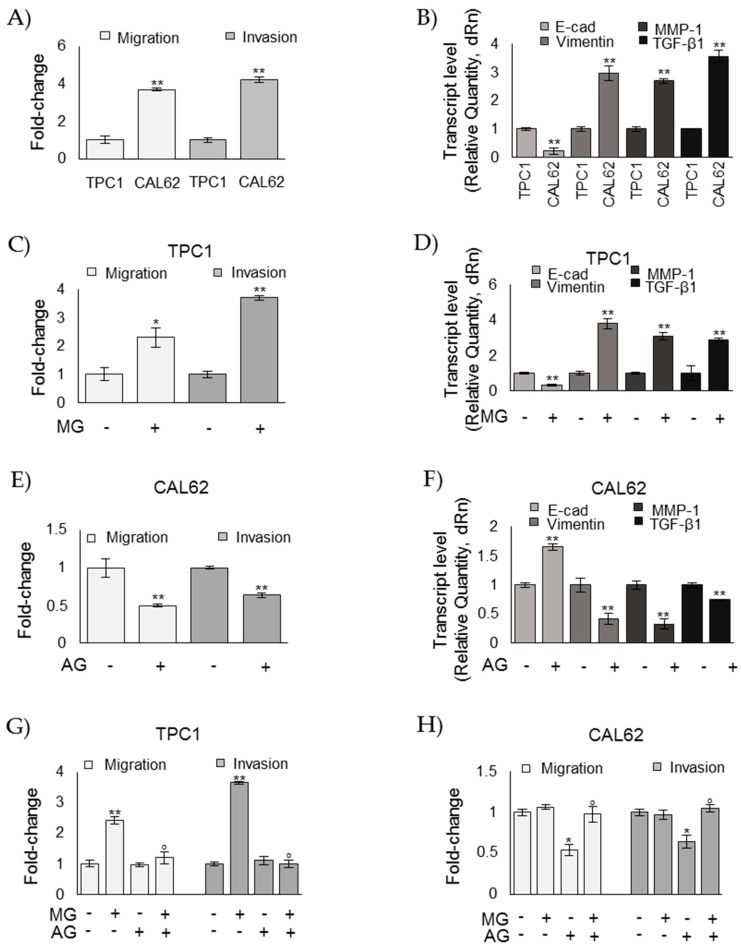
Methylglyoxal (MG) sustains the aggressive phenotype of ATC CAL62 cells. Papillary thyroid cancer (BCPAP and TPC1) and anaplastic thyroid cancer (8505C and CAL62) cells grown to confluence under standard conditions were analyzed by specific assays or real-time PCR to evaluate their migration and invasion capabilities (**A**,**C**,**E**,**G**,**H**) or the mRNA expression of E-cadherin (E-cad), Vimentin, MMP-1, and TGF-β1 (**B**,**D**,**F**), respectively, as described in the Materials and Methods section. The histograms indicate mean ± SD of three different cultures, and each was tested in triplicate. mRNA expression levels were normalized to the amount of an internal control transcript (β-actin). Invasion/migration capabilities as well as gene expression were measured in TPC1 after 5 µM MG administration and in CAL62 cells after 1 mM aminoguanidine (AG), both for 48 h. Invasion/migration were also evaluated after MG and AG cotreatment (**G**,**H**) at the concentrations above reported. The histogram indicates mean ± SD of three different cultures, and each was tested in triplicate. * *p* < 0.05; ** *p* < 0.01 compared to untreated cells; ° *p* < 0.01 compared with MG (**G**) or AG (**H**) exposure alone.

To investigate whether MG was involved in the aggressiveness of ATC cell phenotypes, we next examined the effect of MG administration and AG, a scavenger of free MG [8,39], on TPC1 and CAL62 cell lines, respectively. We evaluated cell migration/invasion and epithelial-to-mesenchymal transition (EMT), which are phenomena typically related to an aggressive behavior [8,40]. In particular, EMT was studied by analyzing the expression of E-cadherin (E-cad)—a typical marker of epithelial cells; vimentin—a typical marker of mesenchymal cells; MMP-1—one of the ten top globally upregulated genes in ATC [8,41]; and TGF-β1, whose overexpression is known to promote migration and invasion in many cancers, including ATC [8,42]. As shown in Figure 4A, CAL62 ATC cells, where MG-H1 accumulated as a result of Glo1 functional depletion, exhibited significantly increased migration and invasion abilities compared with TPC1 PTC. Moreover, CAL62 cells were characterized by decreased expression of E-cad and concurrently increased expression of vimentin, MMP-1, and TGF-β1 compared to TPC1 cells (Figure 4B). MG treatment on TPC1 cells induced migration/invasion (Figure 4C), reduced E-cad expression, and induced vimentin, MMP-1, and TGF-β1 expression (Figure 4D). Accordingly, AG treatment on CAL62 cells decreased migration/invasion (Figure 4E), increased E-cad expression, and reduced vimentin, MMP-1, and TGF-β1 expression (Figure 4F). More importantly, upon MG treatment, AG reverted MG-induced effects on TPC1 cell migration/invasion (Figure 4G), and upon AG treatment, MG reverted AG-induced effects on CAL62 cell migration/invasion (Figure 4H), thus corroborating the protumor effect of MG in TC progression.

**Figure 5:**
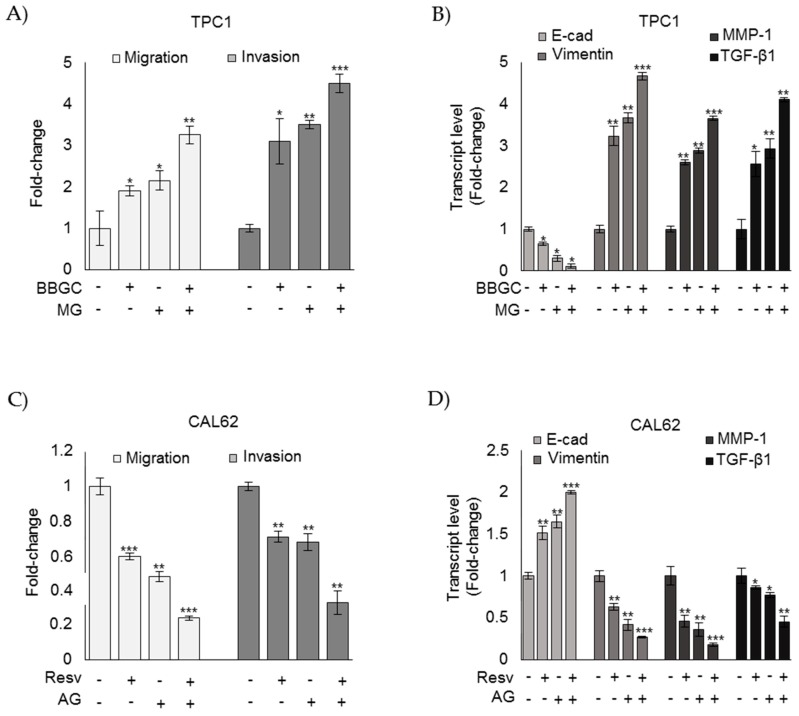
Methylglyoxal (MG) sustains the aggressive phenotype of ATC CAL62 cells. Papillary thyroid cancer TPC1 and anaplastic thyroid cancer CAL62 cells grown to confluence under standard conditions were analyzed by specific assays or real-time PCR to evaluate their migration and invasion capabilities (**A**,**C**) or the mRNA expression of E-cadherin (E-cad), Vimentin, MMP-1, and TGF-β1 (**B**,**D**), respectively, as described in the Materials and Methods section. The histograms indicate mean ± SD of three different cultures, and each was tested in triplicate. mRNA expression levels were normalized to the amount of an internal control transcript (β-actin). Invasion/migration capabilities as well as gene expression were measured in TPC1 after 5 µM MG administration and in CAL62 cells after 1 mM aminoguanidine (AG), both for 48 h. The histogram indicates mean ± SD of three different cultures, and each was tested in triplicate. * *p* < 0.05; ** *p* < 0.01; *** *p* < 0.01.

To further prove that Glo1/MG-H1 axis plays a key role in TC progression, we examined the effect of MG on BBGC treatment in TPC1 cells and that of AG on resveratrol treatment in CAL62 cells. As depicted in Figure 5, in TPC1 cells, Glo1 inhibition by BBGC increased migration and invasion (Figure 5A) and the EMT-associated phenotype at the level of mRNA expression of epithelial or mesenchymal cell markers (Figure 5B) as well as MMP-1 and TGF-β1 expression (Figure 5B) with respect to untreated cells. More importantly, upon BBGC treatment, MG exposure was able to potentiate migration and invasion (Figure 5A), the EMT-associated phenotype, at the level of mRNA expression of epithelial or mesenchymal cell markers (Figure 5B), as well as MMP-1 and TGF-β1 expression (Figure 5B) with respect to cells treated with BBGC or MG alone. Similarly, in CAL62 cells, upon resveratrol treatment, AG exposure was able to further reduce migration and invasion (Figure 5C), the EMT-associated phenotype, at the level of mRNA expression of epithelial or mesenchymal cell markers (Figure 5D) as well as MMP-1 and TGF-β1 expression (Figure 5D) with respect to cells treated with resveratrol or AG alone. Together, these findings support a mechanism whereby Glo1 depletion acts by inducing accumulation of intracellular levels of MG-H1 in ATC cells, thus sustaining the aggressive phenotype of these cells.

### 3.5. MG Sustains the Aggressive Phenotype of ATC CAL62 Cells by Modulating TGF-β1 Secretion and Focal Adhesion Kinase (FAK) Signaling

**Figure 6:**
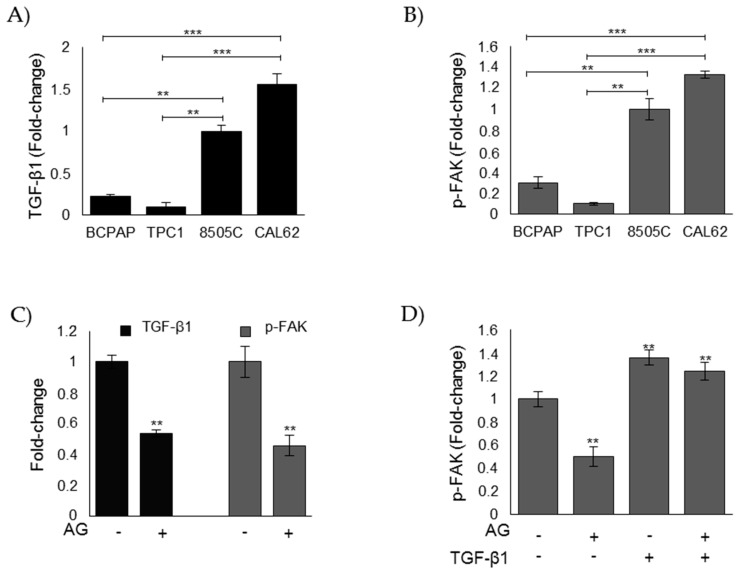
MG sustains the aggressive phenotype of ATC CAL62 cells by modulating TGF-β1 secretion and FAK signaling. Evaluation of (**A**) secreted TGF-β1 and phospho-FAK (p-FAK) (**B**) levels, measured by specific ELISA kits in the culture supernatant or lysate, respectively, of PTC (BCPAP and TPC1) and ATC (8505C and CAL62) cell models; (**C**) levels of TGF-β1 and p-FAK in ATC CAL62 cells treated (+) with 1 mM aminoguanidine (AG). Untreated (−) cells were used as controls. (**D**) Intracellular levels of p-FAK in ATC CAL62 cells treated with 1 mM AG and/or treated with 5 ng/mL TGF-β1 for 48 h. The histograms indicate mean ± SD of three different cultures, and each was tested in triplicate. ** *p* < 0.01, *** *p* < 0.001.

TGF-β1 is a potent mediator of EMT in ATC by activating focal adhesion kinase (FAK) signaling [43]. We here demonstrated that MG-derived dicarbonyl stress increased TGF-β1 expression and dedifferentiation towards an EMT phenotype. Therefore, we firstly investigated the basal level of secreted TGF-β1 and activated p-FAK in ATC CAL62 and 8505C cells as well as PTC B-CPAP and TPC1 cells for comparison. We found that ATC CAL62 and 8505C cells showed markedly higher levels of secreted TGF-β1 (Figure 6A) and activated p-FAK (Figure 6B) than PTC B-CPAP and TPC1 cells. Interestingly, we found higher levels of secreted TGF-β1 and activated p-FAK in CAL65 ATC cells where the highest MG-H1 intracellular accumulation (Figure 3A) and the lowest Glo1 enzymatic activity was observed (Figure 3D). We also found markedly lower levels of secreted TGF-β1 and activated p-FAK in TPC1 PTC cells, where the lowest levels of MG-H1 intracellular accumulation (Figure 3A) and the highest levels of Glo1 enzymatic activity (Figure 3D) were observed. Subsequently, we investigated whether MG-H1 could drive TGF-β1 secretion, and whether this was paralleled by the activation of FAK pathway in ATC CAL62 cells where the major biological effects studied were observed. We found that upon AG treatment, both TGF-β1 levels in the culture supernatants and the levels of activated p-FAK in the lysate of CAL62 cells were significantly reduced compared to untreated cells (Figure 6C), suggesting that MG sustained the aggressive phenotype of ATC CAL62 cells in a mechanism mediated by TGF-β1/FAK signaling. Cotreatment of CAL62 cells with AG and exogenous TGF-β1 unequivocally demonstrated this hypothesis (Figure 6D).

Moreover, a significant, positive correlation was found between MG-H1 and TGF-β1 (Spearman’s correlation coefficient = 0.87, *p* = 0.0020) or MG-H1 and p-FAK (Spearman’s correlation coefficient = 0.77, *p* = 0.0032) levels in protein extracts from ATC (*n* = 5, #1–5) tissues, thus further supporting the results obtained in vitro in ATC CAL62 cells.

### 3.6. Glo1 Depletion and Related Downstream Events are Under the Partial Control of IL-1β in ATC CAL62 Cells

**Figure 7:**
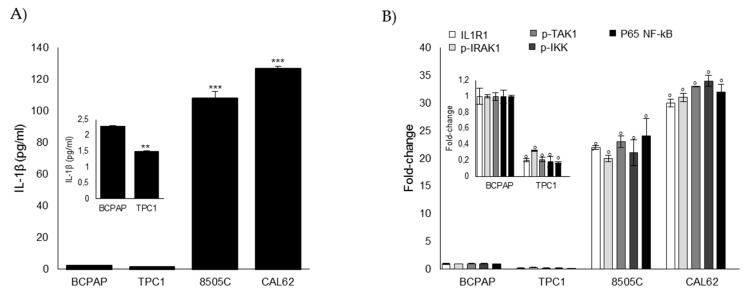
IL-1β signaling in human TC cell lines. PTC (BCPAP and TPC1) and ATC (8505C and CAL62) cell models grown to confluence under standard conditions were analyzed by specific ELISA kits, following the manufacturer’s instructions, to evaluate (**A**) the secreted levels of IL-1β (measured in the medium) and (**B**) levels of IL1 receptor, type I (ILR1) (measured in the cell lysates), phospho-IRAK-1 (p-IRAK1) (a cell-based method), phospho-TAK1 (p-TAK1) (measured in the cell lysates), phospho-IKK (p-IKK) (a cell-based method), and P65 NF-kB (measured in the nuclear extracts). The histograms indicate mean ± SD of three different cultures, and each was tested in triplicate. The inserts represent the original histograms without ATC cells in order to better appreciate changes in PTC cells. ** *p* < 0.01, *** *p* < 0.001, and ° *p* < 0.001.

Experimental and epidemiological studies revealed that chronic inflammation is a feature of ATC [44,45,46]. In particular, ATC shows increased expression of several proinflammatory cytokines (especially of IL-1β) that, via a feedforward autocrine loop, promotes cell invasion in this cancer type [46]. In order to also verify the physiological relevance of IL-1β in our in vitro cell models, we firstly investigated IL-1β secretion and signaling in ATC CAL62 and 8505C cells as well as in PTC B-CPAP and TPC1 cells for comparison. We found that ATC CAL62 and 8505C cells showed markedly higher levels of secreted IL-1β than PTC B-CPAP and TPC1 cells (Figure 7A). Concordantly, IL-1β signaling was significantly more active in ATC cells than in PTC cells, as indicated by the increase in the expression of IL-1 receptor type I (ILR1) [47,48], phospho-IL-1R-associated kinase (IRAK1), phospho-IRAK1, phospho-TAK1, and nuclear p65-NF-kB [48] (Figure 7B).

**Figure 8:**
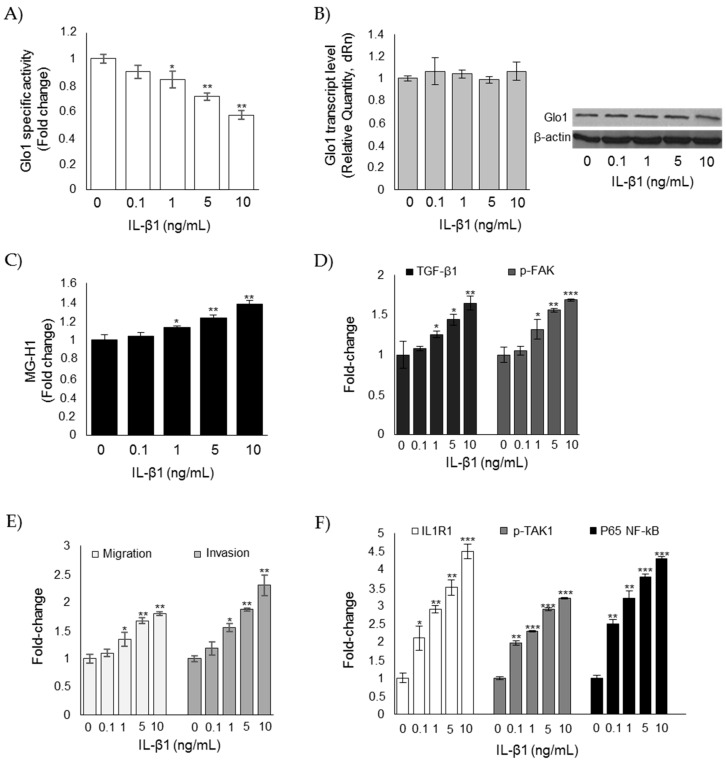
Glo1 depletion and related downstream events are under the partial control of IL-1β in CAL62 cells. Effects of IL-1β on (**A**) Glo1 enzyme activity; (**B**) Glo1 transcript and protein levels; (**C**) intracellular levels of MG-H1; (**D**) TGF-β1 and p-FAK levels, measured in the culture supernatant or lysate of CAL62, respectively; (**E**) migration and invasion capabilities; and (**F**) IL-1β signaling, evaluated by the levels of IL1 receptor type I (ILR1) (measured in the cell lysates), phospho-TAK1 (p-TAK1) (measured in the cell lysates), and P65 NF-kB (measured in the nuclear extracts). Western blots are representative of three different cultures, each tested in triplicate. β-actin was used as internal loading control for WB normalization. Histograms indicate mean ± SD of three different cultures, each tested in triplicate. * *p* < 0.05, ** *p* < 0.01, and *** *p* < 0.001 compared to untreated cells.

Very recently, it has been described that Glo1 functionality is impaired by IL-1β [49]. To understand whether the decrease in Glo1 enzyme activity observed in our model was due to IL-1β, CAL62 cells were exposed to several concentrations of this potent, proinflammatory cytokine, and Glo1 enzymatic activity was measured. As shown in Figure 8A, Glo1 enzymatic activity, but not Glo1 transcript or protein levels (Figure 8B), decreased in response to IL-1β stimulation in a dose-dependent manner, and this was paralleled by an increase of MG-H1 intracellular levels (Figure 8C), TGF-β1/FAK signaling (Figure 8D), as well as migration and invasion (Figure 8E). As expected, IL-1β stimulation also potentiated IL1R1-mediated signaling (as shown by IL1R1), phospho-TAK1, and p65 NF-kB increased expression (Figure 8F).

**Figure 9:**
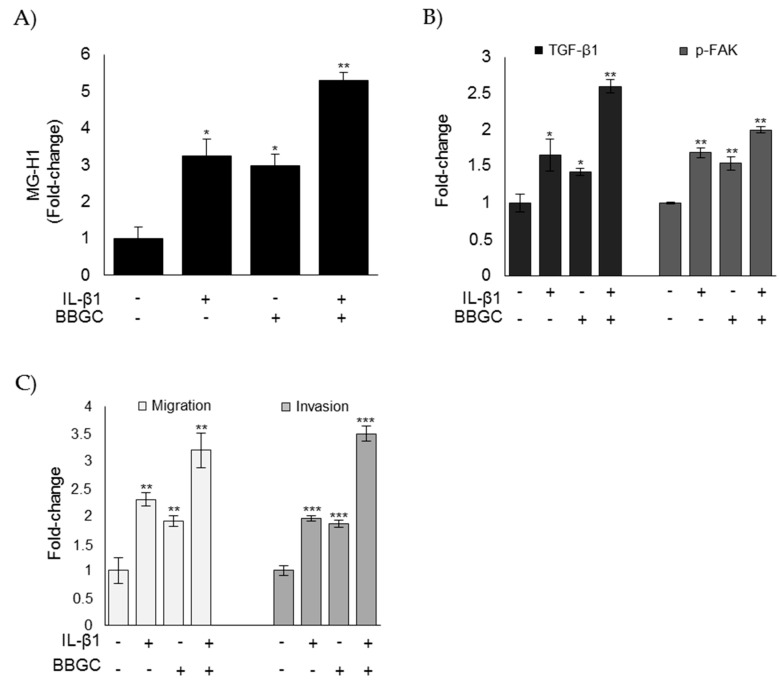
IL-1β partially sustains the aggressive phenotype of CAL62 cells via a novel mechanism mediated by Glo1 inhibition, which drives MG-H1 accumulation and, in turn, activates TGF-β1-mediated FAK signaling. Effects of Glo1 inhibition, under IL-1β administration, on (**A**) intracellular levels of MG-H1, evaluated by ELISA; (**B**) TGF-β1 and p-FAK levels, measured in the culture supernatant or lysate of CAL62 cells, respectively, by ELISA; and (**C**) migration and invasion capabilities, evaluated by specific assays. Histograms indicate mean ± SD of three different cultures each tested in triplicate. * *p* < 0.05, ** *p* < 0.01, and *** *p* < 0.001 compared to untreated cells.

Moreover, upon IL-1β administration, Glo1 inhibition by BBGC potentiated MG-H1 intracellular accumulation (Figure 9A), activation of TGF-β1/FAK signaling (Figure 9B), as well as migration and invasion (Figure 9C). These results suggested that IL-1β, at least in part, sustained the aggressive phenotype of CAL62 cells (in terms of migration and invasion) via a novel mechanism mediated by Glo1 inhibition, which drove MG-H1 accumulation and, in turn, activated TGF-β1-mediated FAK signaling. However, involvement of other pathways impairing Glo1 activity might not be excluded.

**Figure 10:**
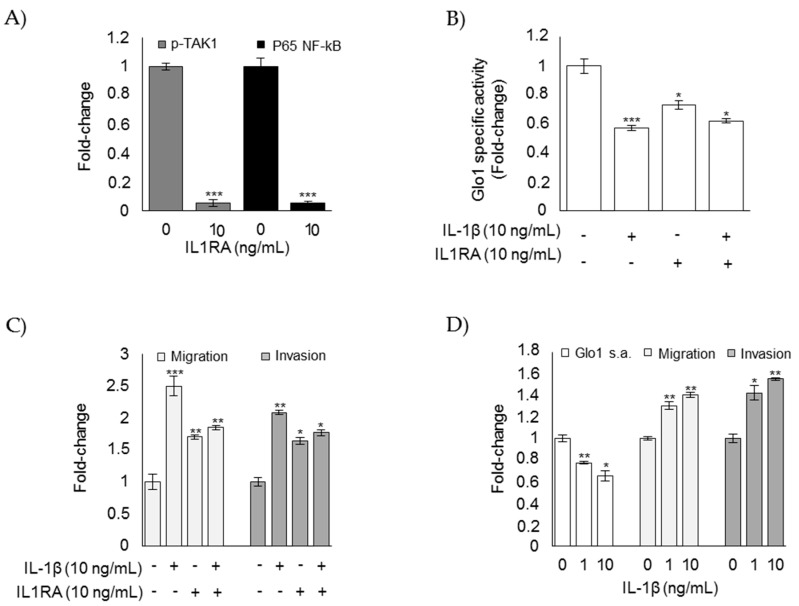
The effect of a specific IL-1 receptor antagonist (IL1RA) administrated to CAL65 anaplastic thyroid cancer cells for 3 h alone (**A**) or in combination with IL-1β (**B,C**) on (**A**) phospho-TAK1 (p-TAK1) (measured in the cell lysates) and P65 NF-kB (measured in the nuclear extracts) by ELISA; (**B**) Glyoxalase 1 (Glo1)-specific activity, measured by a spectrophotometric assay; and (**C**) migration and invasion capabilities, evaluated by specific assays. (**D**) Effect of the long-term exposure (2 weeks) of TPC1 papillary thyroid cancer cells to IL-1β on Glo1 specific activity (s.a.), migration, and invasion. Histograms indicate mean ± SD of three different cultures each tested in triplicate. * *p* < 0.05, ** *p* < 0.01, and *** *p* < 0.001 compared to untreated cells.

To further prove the contribution of IL-1β in driving loss of Glo1 activity and the subsequent mesenchymal-invasive phenotype, we either treated CAL62 ATC cells with a specific IL-1β receptor antagonist (IL1RA) [50] or chronically (2 weeks) exposed TPC1 PTC cells to IL-1β in independent experiments. As expected, IL-1β signaling was significantly reduced in CAL62 cells after IL1RA treatment (Figure 10A). Moreover, IL1RA only partially reverted Glo1 activity (Figure 10B) and the mesenchymal-invasive phenotype of CAL62 cells (Figure 10C). Finally, long-term exposure of TPC1 cells to IL-1β induced, although significant, only a moderate decrease in Glo1 activity and an increase in the mesenchymal-invasive phenotype (Figure 10D), thus confirming the partial involvement of IL-1β in the observed biological phenomena.

### 3.7. Nutraceutical Resveratrol Affects the CAL62 Cell Aggressive Phenotype by Reducing Migration and Invasion through the Inhibition of IL-1β-Driven, Glo1/MG-H1-Mediated TGF-β1/FAK Signaling

**Figure 11:**
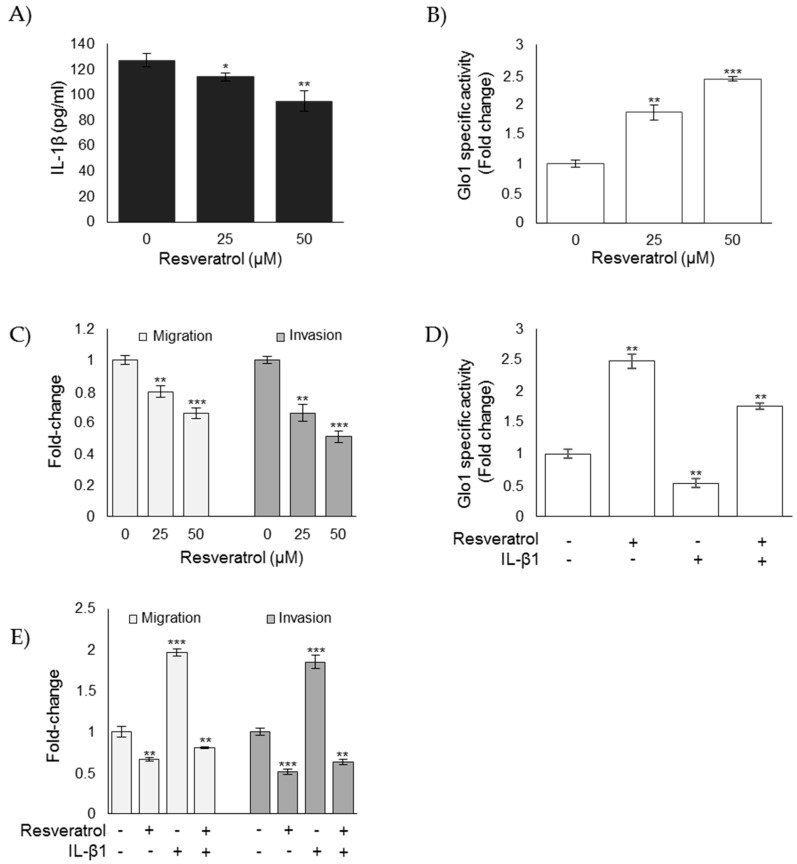
Resveratrol affects the CAL62 cell aggressive phenotype by reducing migration and invasion through the inhibition of IL-1β-driven, Glo1/MG-H1-mediated TGF-β1/FAK signaling. Effect of resveratrol on (**A**) IL-1β levels, evaluated by an ELISA kit in cell culture medium; (**B**) Glo1-specific activity, measured by spectrophotometry; and (**C**) migration and invasion capabilities, evaluated by specific assays. Effect of IL-1β, under resveratrol administration, on (**D**) Glo1 enzyme activity and (**E**) migration and invasion capabilities. Histograms indicate mean ± SD of three different cultures, and each was tested in triplicate. * *p* < 0.05, ** *p* < 0.01, and *** *p* < 0.001.

Experimental evidence suggested that resveratrol-treated ATC cells showed remarkable growth arrest and extensive apoptosis [27,29,51], and that it was able to reduce IL-1β expression in either in vivo or ex vivo human noncancerous models [52,53] or melanoma cells [54]. Moreover, resveratrol was able to upregulate Glo1 expression and activity [38]. Therefore, we wanted to investigate whether resveratrol could reverse the aggressive phenotype of CAL62 ATC cells in terms of EMT, migration, and invasion by inhibiting IL-1β-dependent downstream pathways involving Glo1/MG-H1- and TGF-β1-mediated FAK signaling. We found that resveratrol significantly inhibited IL-1β expression (Figure 11A) and increased Glo1 activity (Figure 11B) in a dose-dependent manner compared to untreated control cells. Moreover, this was associated with a reversal of migratory and invasive potential (Figure 11C). More importantly, following resveratrol administration, IL-1β treatment reversed, at least in part, an increase in Glo1 activity (Figure 11D) and a decrease in migration/invasion (Figure 11E).

## 4. Discussion

A cancer-promoting role of MG-derived dicarbonyl stress has been recently described in colorectal cancer [24,26]. In line with these results, we have here demonstrated, for the first time, a protumorigenic role of MG-derived dicarbonyl stress also in ATC, where it sustained a metastatic phenotype through the control of EMT, migration, and invasion—traits that are essential for metastasis. Hence, the role of MG and MG-derived AGEs, long considered potent cytotoxic molecules, is emerging to be specific to cancer type and stage. Therefore, caution must be taken when inducing MG accumulation as an anticancer strategy, at least in some malignancies.

EMT is a transdifferentiation process in which epithelial cells switch to a mesenchymal phenotype by losing their polarity and acquiring increased motility [8] and invasive abilities, which is further fostered by the activity of MMP family proteins. EMT is a crucial prerequisite for the acquisition of metastatic potential in cancer cells [8], including ATC [55]. However, the processes controlling EMT in malignant cells are still emerging. Here, we demonstrated that MG, through MG-H1, sustained the metastatic phenotype of ATC CAL62 cells via the control of EMT, thus further extending knowledge of the factors contributing to EMT development in cancer cells. Moreover, despite that MG dicarbonyl stress has recently been associated with the aggressiveness of some cancers [24,25], the molecular mechanisms underlying this effect have not been clarified yet, particularly regarding MG-driven metastatic phenotype acquisition. We here show that MG, through MG-H1, sustains the aggressive phenotype of ATC CAL62 cells in a pathway mediated by TGF-β1/FAK signaling, which is very likely in an autocrine loop as MG-H1 is able to control TGF-β1 secretion. In good accordance with and supporting this hypothesis is the fact that in ATC-derived cells, TGF-β1 signaling modulates EMT in association with an upregulation of TGFβ receptor 1 (TGFBR1) expression, and inhibition of TGFBR1 in these cells can reverse EMT [56].

TGF-β is a cytokine that plays a fundamental role in various cellular functions. However, deregulation of the TGF-β pathway can lead to various pathological conditions, including cancer. Although studies have demonstrated the tumor suppressive role of TGF-β during the early stages of tumor development, it switches to a tumor promoter during the advanced metastatic stages of cancer [57], including ATC [43], where it acts as a tumor-promoting factor associated with EMT induction, increased invasion, extrathyroid extension, and lymph node metastases [43]. TGF-β is a major inducer of EMT in many neoplastic cell types. However, the molecular mechanisms by which TGF-β induces EMT in advanced stages of cancer are poorly characterized. TGF-β1, the most ubiquitous and best characterized isoform, promotes tumor progression and metastasis in advanced cancers via both Smad-dependent pathways and Smad-independent pathways. Here, in ATC CAL62 cells, we demonstrated that TGF-β1 promoted migration and invasion, via FAK signaling, under MG-H1 control. Thus, we identified a novel mechanism in TGF-β1 signaling [43]. FAK is a critical substrate that regulates several cellular events including migration and invasion through its dual function as a kinase and scaffolding protein [46]. FAK autophosphorylation leads to the recruitment of Src, and this complex signals to downstream effectors, including pro-invasive genes, such as MMPs [46]. We observed that MMP1 expression levels increased in ATC cells to promote invasion, suggesting the involvement of this MMP in sustaining the aggressive phenotype of ATC, which was in agreement with a similar role for another MMP (MMP9) in the same malignancy [46].

We also demonstrated that the accumulation of MG-H1 in ATC tumors was consequent to a decreased functionality of Glo1, the major enzymatic defense against MG-mediated glycation [36], thus suggesting a tumor-suppressing role for this protein in ATC. In fact, when further impaired by BBGC, Glo1 was able to potentiate ATC CAL62 cell aggressiveness, while, when activated by resveratrol, Glo1 induced the rescue of the ATC cell phenotype. Hence, the traditional role of Glo1 as a tumor promoting factor [1,8,16,17,18,19,20,31] appears to complement an emerging, novel role of this metabolic enzyme as a tumor suppressor [24,25,26]. The cancer-specific role of Glo1 suggests the need of further studies on a case-by-case basis.

Finally, we demonstrated that Glo1 functional impairment was, at least in part, dependent on IL-1β—the main proinflammatory cytokine that positively regulates ATC invasion [46]—providing a novel, Glo1/MG-H1-dependent pathway through which IL-1β-mediated inflammation promoted invasion of ATC cells [44,45,46]. Intriguingly, we found that an IL-1β-dependent decrease in Glo1 enzyme activity was not paralleled by Glo1 mRNA and protein expression decreases. This suggested that IL-1β signaling, activated in ATC CAL62 cells where it ultimately leads to NF-kB activation, might induce the transcription of genes and the consequent synthesis of proteins that somehow led to Glo1 inactivation, including possibly those regulating the production/scavenging of reactive oxygen species or inducing post-translational modifications. This deserves further investigation. Besides, it has been reported that Glo1 can be affected by reactive oxygen species [6,9], phosphorylation, nitrosylation, and glutathionylation [1]. It is known that MG, directly or through AGEs, acts as a proinflammatory agent [1]. In the present study, we found that IL-1β, through the negative control of Glo1, induced MG-H1 accumulation. We also did not exclude that a vicious cycle may have occurred in ATC tissues, such that local systemic inflammation decreased Glo1 activity and resulted in AGE accumulation, which, in turn, was responsible for the local inflammation that perpetuated the decreased Glo1 functionality.

Collectively, our results define a novel mechanism based on the IL-1β/Glo1/MG-H1/TGF-β1/FAK axis in the molecular etiology of ATC, further extending our knowledge of the general mechanisms underlying the ATC phenotype. Identification of the IL-1β/Glo1/MG-H1/TGF-β1/FAK axis in ATC cell aggressiveness suggests that IL-1β and Glo1, which orchestrate the mechanism, may represent novel potential therapeutic targets for ATC, which remains one of the most lethal human cancers [46]. In this regard, it was significant we showed that resveratrol, a polyphenolic phytoalexin that occurs naturally in many plant species including grapes and berries, was able, by inhibiting IL-1β and activating Glo1, to reduce EMT, migration, and invasion in ATC cells, opening new avenues of preclinical/clinical investigation for the use of this nutraceutical in the chemoprevention and/or treatment of ATC patients [27]. Besides, particular attention has converged on plant-derived, natural bioactive compounds, which have demonstrated beneficial effects on the prevention of many diseases [58], including some tumors, such as ATC [27,29,51]. Increased attention has also been driven by the introduction of new techniques (nanocarries and nanoparticles), which can induce/increase the bioefficacy and bioavailability of nutraceuticals in vivo, and the fact that nutraceuticals are safe [29] and low-cost.

## 5. Conclusions

In conclusion, we demonstrated that MG, via MG-H1, sustained a metastatic phenotype of ATC CAL62 cells by controlling EMT, migration, and invasion in a novel mechanism that involved TGF-β1/FAK signaling and was driven by the IL-1β/Glo1 axis. Thus, we provided valuable new insights into the pathogenesis of ATC and novel options for the development of preventive and therapeutic strategies.

## Figures and Tables

**Table 1:** Patients’ characteristics.

Characteristics	^1^ PTC (*n* = 5) *	^2^ ATC (*n* = 5) *
^3^ Age (y)	36 ± 5.25	69 ± 17.78
Sex		
Female	3	4
Male	2	1
^3^ Diameter (cm)	1.92 ± 0.85	5.62 ± 0.89
Histology pTNM		
T1	3	0
T2	1	0
T3	1	0
T4	0	5
N0	3	0
N1	2	5
N2	0	0
Nx	0	0
M0	4	0
M1	1	0
Mx	0	5

^1^ PTC = papillary thyroid cancer; ^2^ ATC = anaplastic thyroid cancer; ^3^ Means ± SD, * the characteristics of this cohort refer to patients whose samples were used for immunohistochemistry (IHC), in particular #16–20 PTC and #11–15 ATC. Biochemical assays were performed using samples from different patients (#6–10 PTC and #1–5 ATC). pTNM = pathologic tumor and node stage.

**Table 2:** Primer sequences.

Gene	Forward (5′-3′)	Reverse (5′-3′)
Glo1	CTCTCCAGAAAAGCTACACTTTGAG	CGAGGGTCTGAATTGCCATTG
E-cadherin	TTGCGGAAGTCAGTTCAG	CAGAGCCAAGAGGAGACC
Vimentin	GCACACAGCAAGGCGATGG	GGAGCGAGAGTGGCAGAGG
MMP-1	TGTCACACCTCTGACATTCACCAA	AAATGAGCATCCCCTCCAATACCT
TGF-β1	GGCGACCCACAGAGAGGAAATAG	AGGCAGAAATTGGCGTGGTAGC
β-actin	CACTCTTCCAGCCTTCCTTCC	ACAGCACTGTGTTGGCGTAC

## Supplementary Materials

The following are available online at https://www.mdpi.com/2073-4409/8/6/547/s1, Figure S1: Biochemical evidence of BBGC and resveratrol effectiveness on Glo1 enzyme activity.
