# Supplementary material for: Methylglyoxal Acts as a Tumor-Promoting Factor in Anaplastic Thyroid Cancer
Source: Cells. 2019 Jun 6;8(6):547. doi: 10.3390/cells8060547 (PMC6627963; doi:10.3390/cells8060547)
Supplement: Supplementary file 1 [file cells-08-00547-s001.pdf]

A)

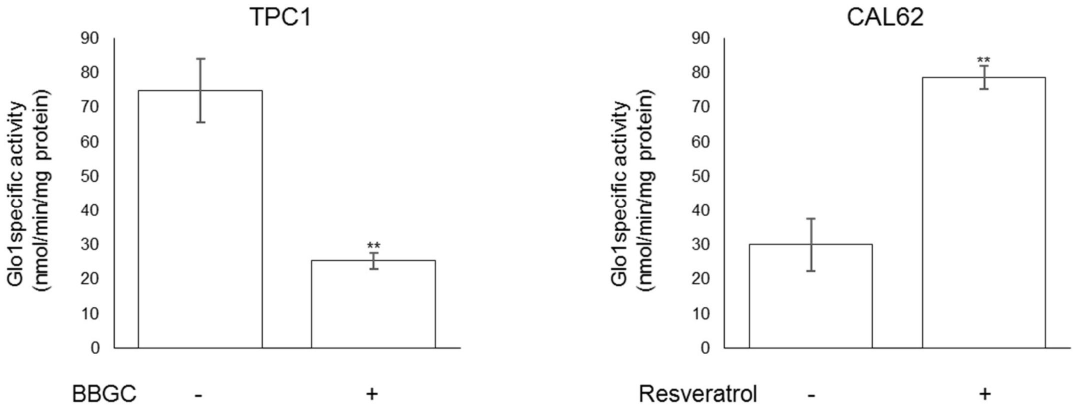

Figure S1. Biochemical evidence of S-p-Bromobenzylglutathione cyclopentyl diester (BBGC) and Resveratrol effectiveness on Glyoxalase 1 (Glo1) enzyme activity. (**A**) Effect of BBGC on glo1 specific activity in a papillary thyroid cancer cell line (TPC1); (**B**) effect of Resveratrol on an anaplastic thyroid cancer cell line (CAL62). Glo1 specific activity was measured by spectrophotometry as described in Materials and Methods. Histograms indicate mean ± SD of three different cultures, and each was tested in triplicate. **$p < 0.01$

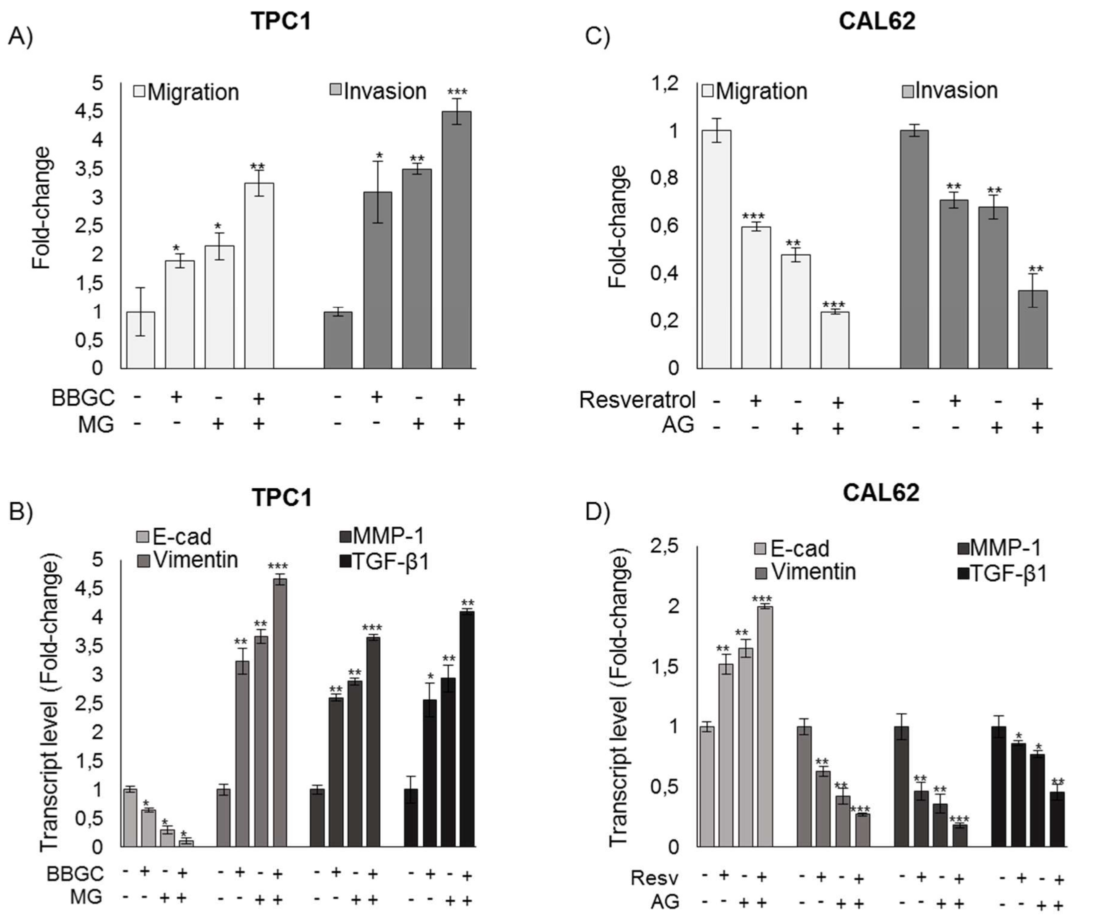

Figure S2. Effect of Methylglyoxal (MG), upon BBGC treatment, on (**A**) migration and invasion or (**B**) E-cadherin (E-cad), vimentin, MMP-1 and TGF-β1 expression in papillary thyroid cancer TPC1 cells. Effect of aminoguanidine (AG), upon Resveratrol treatment, on (**C**) migration and invasion or (**D**) E-cad, vimentin, MMP-1 and TGF-β1 expression in anaplastic thyroid cancer CAL62 cells. Migration and invasion capabilities were measured by using specific assay as described in Materials and Methods. Gene transcript levels were evaluated by real-time PCR. Histograms indicate mean ± SD of three different cultures, and each was tested in triplicate. $^*p < 0.05$, $^{**}p < 0.01$, $^{***}p < 0.001$
